# Effects of concomitant combination of SGLT‐2 inhibitor and GLP‐1 receptor agonist on renal outcomes in T2D with eGFR below 30 and macroalbuminuria: A case series

**DOI:** 10.1002/ccr3.4022

**Published:** 2021-03-09

**Authors:** Nitesh D. Kuhadiya, Israa Mahmood

**Affiliations:** ^1^ Division of Endocrinology, Diabetes & Metabolism Renown Health, Reno & DECON (Diabetes & Endocrine Center of Nevada) Reno NV USA; ^2^ Division of Endocrinology, Diabetes & Metabolism Renown Health Reno NV USA

**Keywords:** eGFR below 30, GLP‐1RA, macroalbuminuria, renal protection, SGLT‐2 inhibitor, type 2 diabetes

## Abstract

Renal protection is likely to be a class effect of SGLT‐2 inhibitors and GLP‐1RA. When used simultaneously, there may be a synergistic effect. Both agents are also safe to use in high renal risk patients (eGFR between 21 and 30 mL/min/1.73m^2^)

## Graphical abstract

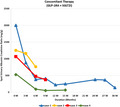

## 1 INTRODUCTION

We hypothesized that the concomitant combination therapy of SGLT‐2 (Sodium‐glucose cotransporter‐**2**) inhibitor and Glucagon‐like peptide‐1 receptor agonist (GLP‐1RA) provides renal protection in adults with type 2 diabetes (T2D) even if estimated GFR (eGFR) is below 30 mL/min/1.73 m^2^. We hereby describe four consecutively treated high renal risk patients with T2D with eGFR between 21 and 30 mL/min/1.73 m^2^ with KDIGO Stage G4A3 and NIH Stage 4 CKD with concomitant SGLT‐2 inhibitor and GLP‐1RA. Cases 1 and 4 received daily empagliflozin (10 mg) while cases 2 and 3 received daily dapagliflozin (5 mg) and ertugliflozin (15 mg), respectively. All four cases received weekly dulaglutide 1.5 mg. The mean Urinary Albumin Creatinine Ratio (UACR) at baseline was 1211 ± 304 mg/g. Cases 1, 2, and 3 had nephrotic range proteinuria while case 4 had UACR of 527 mg/g. There was a regression of mean UACR of all four cases by 16%, 65%, and 77% at 3, 6, and 9 months of treatment, respectively, compared to baseline. None of the four cases reported end‐stage kidney disease (dialysis, transplantation, or a sustained eGFR of <15 mL/min/1.73 m^2^), a doubling of the serum creatinine level, or death from renal or cardiovascular causes during a follow‐up period ranging from 6 to 30 months.

The arrival of SGLT‐2 inhibitors has spelt a revolution in the way we manage our adults with type 2 diabetes (T2D) today.^1^, ^2^ These agents have shown benefits in reducing cardiovascular and renal risk ^1^ in addition to glycemic control, weight loss, and antihypertensive effects. Diabetes is the leading cause of kidney disease and kidney failure in the United States.^1^ Chronic kidney disease (CKD) or diabetic kidney disease (DKD) is progressive, irreversible and often goes undetectable.^1^ CREDENCE trial showed that Canagliflozin (100 mg per day dose) when used in adults with diabetic kidney disease (DKD) with eGFR of >30 mL/min/1.73 m^2^ and median urinary albumin creatinine ratio (UACR) of 927 mg/day lead to reduction in primary composite end point of end‐stage kidney disease (dialysis, transplantation, or a sustained estimated eGFR of <15 mL/min/1.73 m^2^), a doubling of the serum creatinine level, or death from renal or cardiovascular causes.^2^ Recently, DAPA‐CKD showed similar benefits with dapagliflozin in patients with and without T2D with eGFR as low as 25 mL/min/1.73 m^2^ ^3^ A study comparing dulaglutide with insulin glargine (AWARD‐7) in which 29% of the participants had a eGFR of <30 mL/min/1.73 m^2^ showed reduced decline in eGFR and increased reduction in albuminuria comparing to the insulin group in post hoc analysis.^4^ However, the effects of concomitant SGL‐2 inhibitor with the GLP1 RA on renal outcomes have not been reported to the best of our knowledge in patients with DKD with eGFR below 30 (between 21 and 30 mL/min/1.73 m^2^). We hereby describe four such case reports.

## 2 METHODS

We hypothesized that concomitant use of SGLT‐2 inhibitors and GLP‐1 RA provide renal protection in patients with eGFR below 30 mL/min/1.73 m^2^. We implemented this strategy at our clinic and retrospectively collected the required follow‐up data of 4 consecutively treated patients who had at least one baseline HbA1c, UACR, Basic Metabolic panel (BMP) and at least a total of two follow‐up HbA1c, UACR, and Basic Metabolic Panel(BMP) at approximately 3‐month interval. The main methods used include measuring urinary albumin creatinine ratio (UACR), HbA1c, and Basic Metabolic Panel at approximately every 3 months follow‐up period. We hereby describe these four individual case reports.

## 3 RESULTS

**FIGURE 1:**
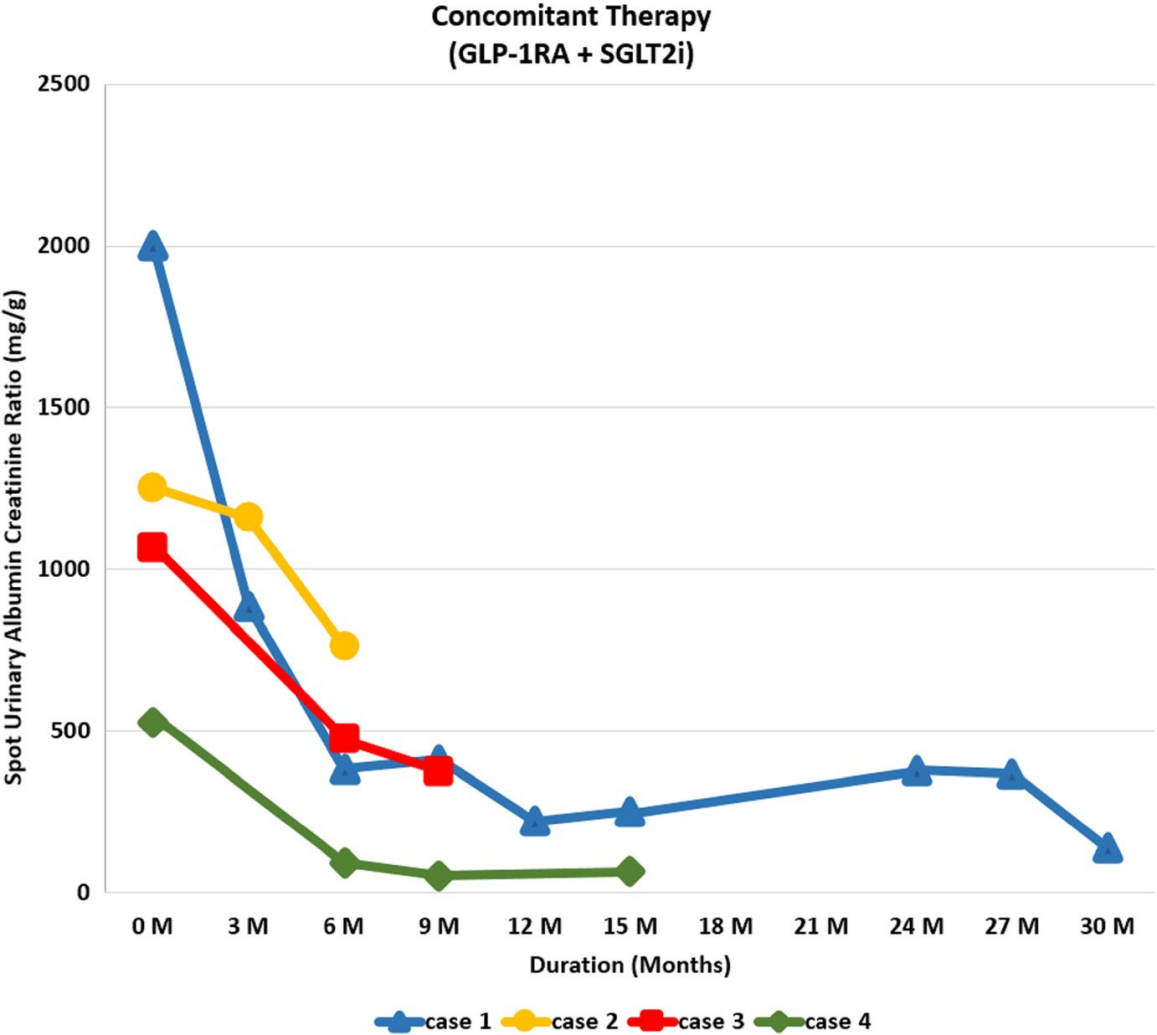
Graph showing trend of spot urinary albumin creatinine ratio of cases 1‐4 on concomitant therapy

**FIGURE 2:**
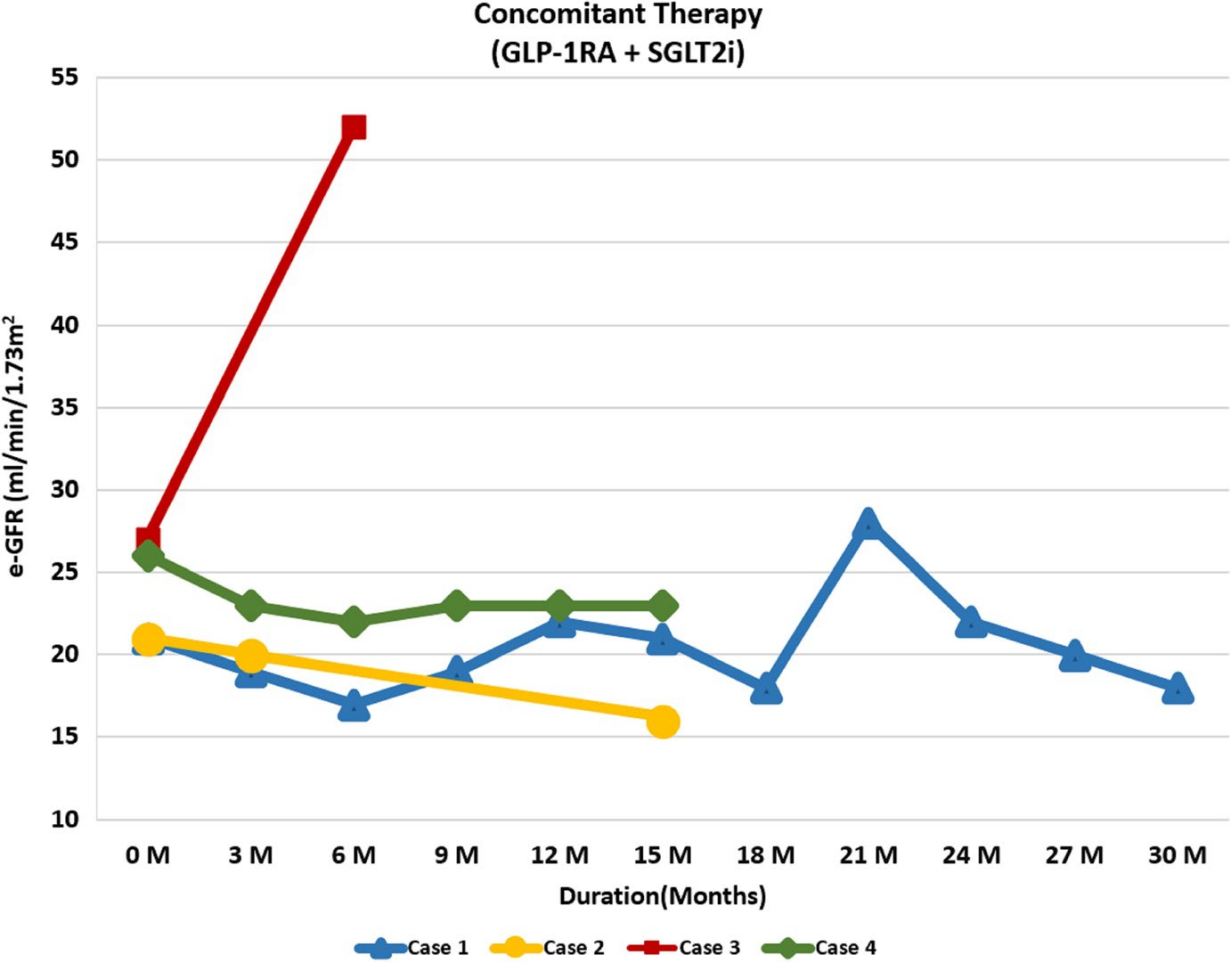
Graph showing trend of eGFR in cases 1‐4 on concomitant therapy

**FIGURE 3:**
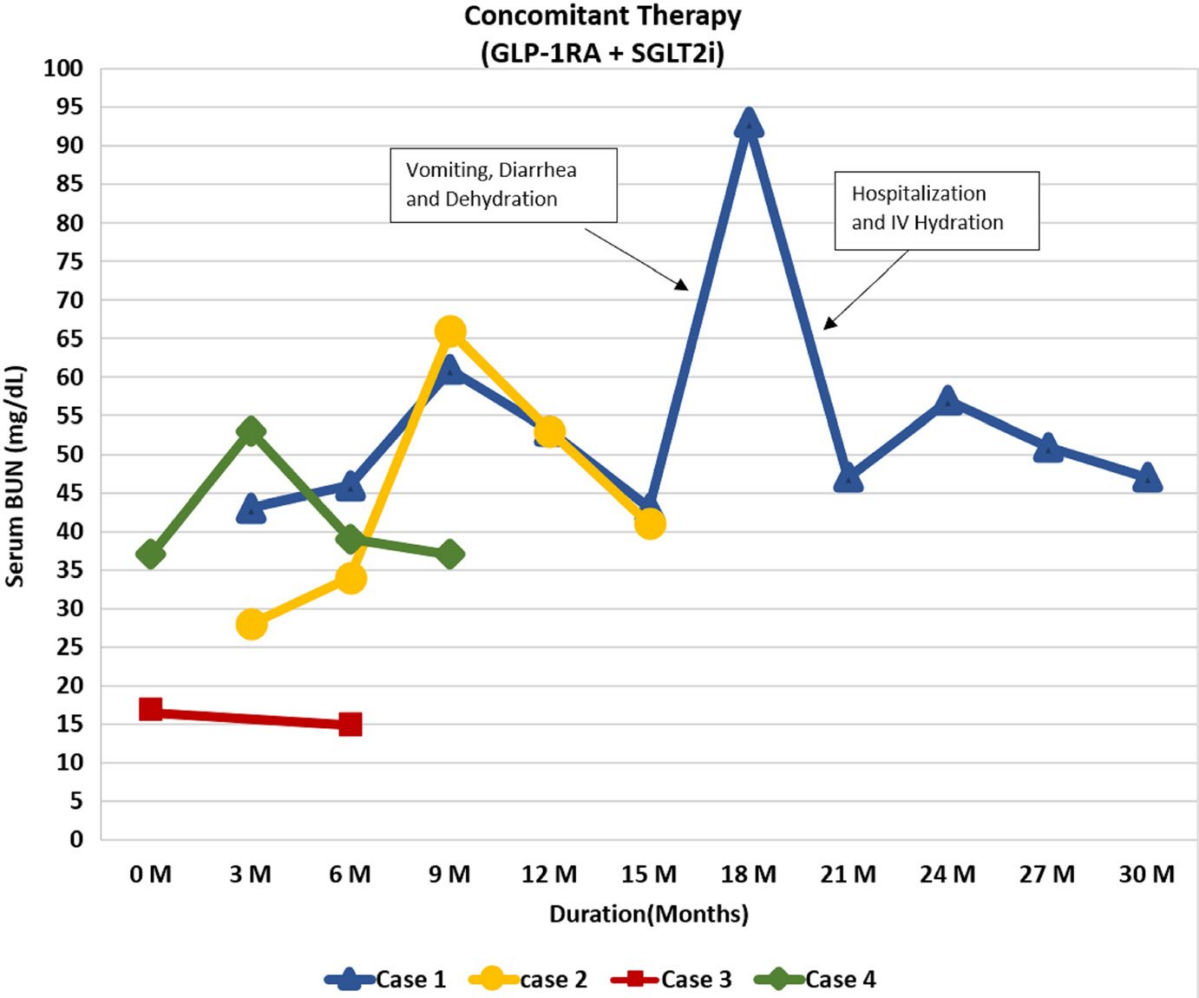
Graph showing trend of serum BUN in cases 1‐4 on concomitant therapy

**FIGURE 4:**
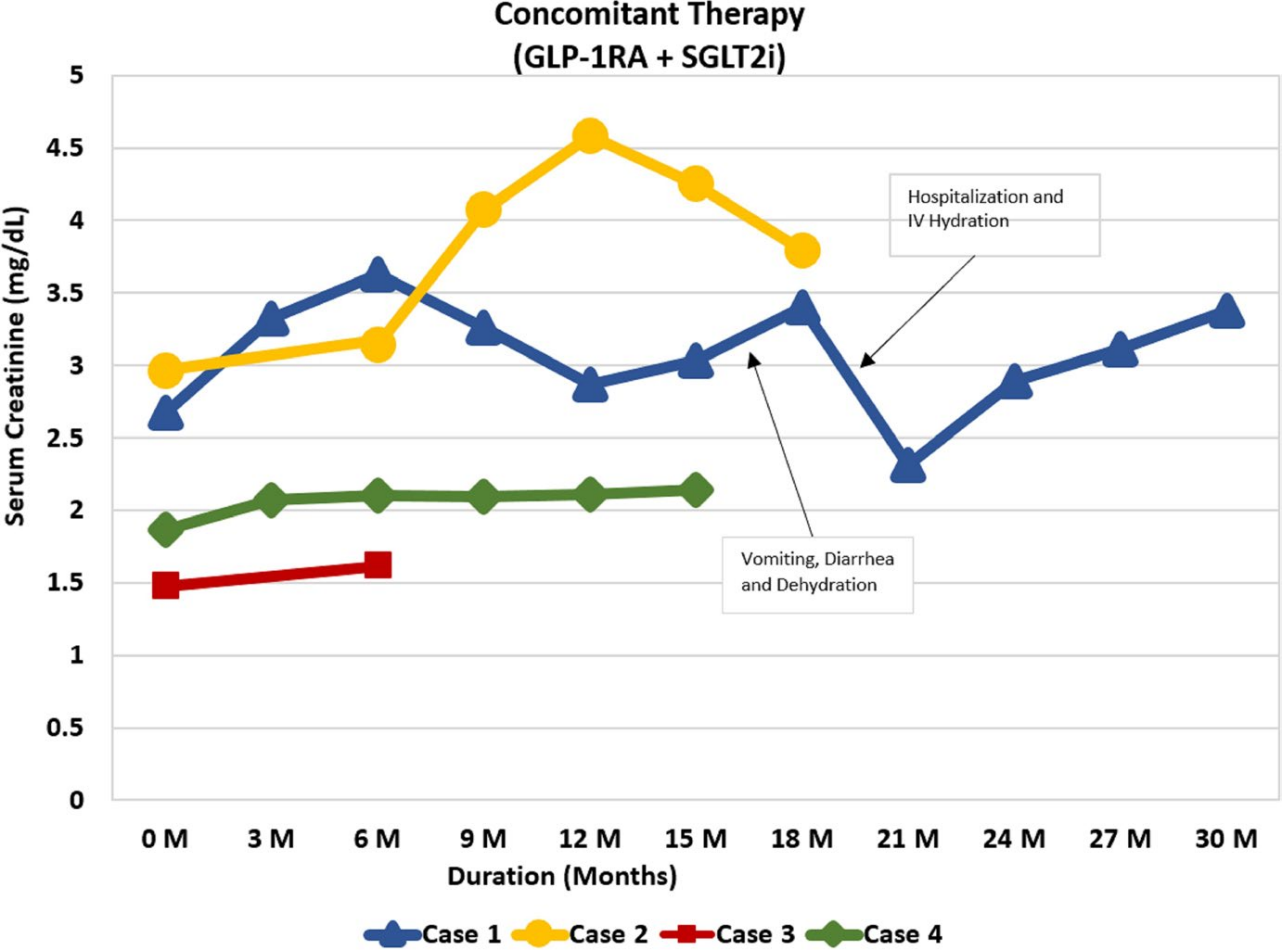
Graph showing trend of serum creatinine in cases 1‐4 on concomitant therapy

Please also refer to Table 1, Figures 1, 2, 3, 4 for more details.

**TABLE 1:** Clinical Characteristics of Cases 1‐4

	Case 1	Case 2	Case 3	Case 4
Age(y)	65	69	62	74
Gender(M/F)	M	M	M	F
HbA1C (%)	11.4	6.1	13	7.8
Duration of T2D(yrs)	6	7	4	8
Weight (Kg)	102.5	90	87.1	71.6
BMI (kg/m^2^)	33.37	26.9	28.34	27.95
BP (mm Hg) PR (bpm)	126/80 78	132/64 60	148/78 82	98/60 86
HTN (Y/N)	Y	N	Y	Y
Dyslipidemia (Y/N)	Y	N	Y	Y
ACE/ARB (Daily Dose)	Valsartan (80 mg)	Lisinopril (20 mg)	Losartan (100 mg)	Losartan (100 mg)
Statin (Daily dose)	Rosuvastatin (40 mg)	Atorvastatin (20 mg)	Atorvastatin (20 mg)	Rosuvastatin (40 mg)
Concomitant Therapy(A + B)				
A)GLP‐1RA (Weekly Dose)	Dulaglutide (1.5 mg)	Dulaglutide (1.5 mg)	Dulaglutide (1.5 mg)	Dulaglutide (1.5 mg)
B)SGLT2i (Daily dose)	Empagliflozin (10 mg)	Dapagliflozin (5 mg)	Ertugliflozin (15 mg)	Empagliflozin (10 mg)
Concomitant Therapy Duration (GLP‐1RA + SGLT2i) (months)	30	6	9	15
Basal Insulin(Y/N)	Y	N	Y	Y
Prandial Insulin(Y/N)	Y	N	Y	Y
TZD (Y/N)	Y	N	N	N
NIH Staging CKD	Stage 4	Stage 4	Stage 4	Stage 4
KDIGO Staging	G4A3	G4A3	G4A3	G4A3
Baseline UACR (mg/g)	2000	1253	1065	527
Baseline C02(mmol/L) (20‐30mmol/L)	23	25	28	24

Abbreviations: ACEI/ARB, Angiotensin‐converting enzyme inhibitors/Angiotensin II Receptor Blocker; BP, Blood Pressure; GLP‐1RA, Glucagon‐Like Peptide 1 Receptor Agonist; HTN, Hypertension; KDIGO, Kidney Disease Outcome Quality Initiative; NIH, National Institute of Health; PR, Pulse Rate; SGLT‐2 Inhibitor, Sodium‐glucose Cotransporter‐**2** inhibitor; TZD, Thiazolidinediones; UACR, Urin Albumin to Creatinine Ratio.

### 3.1 Case 1

68‐year‐old Caucasian gentleman with six‐year history of T2D presented to our clinic with HbA1C of 11.4%, UACR of 2000 mg/g, a eGFR of 21 mL/min/1.73 m^2^, BUN of 43 mg/dL, creatinine of 2.67 mg/dL, NIH Stage 4 CKD and KDIGO Stage G4A3. His vitals are shown in Table 1, and electrolyte levels were within normal limits. At the time of his first visit with us, his T2DM was managed with Glimepiride 4mg two times daily, 30mg of Pioglitazone daily, 98 units of Insulin Glargine (300 units/mL) at bedtime, and 30 units of Insulin Aspart (100 units/mL) before breakfast, lunch, and dinner. He was on rosuvastatin 40 mg and valsartan 80mg daily to treat his dyslipidemia and HTN, respectively. Empagliflozin 10 mg daily was initiated along with Exenatide ER SC once weekly. He was on Exenatide ER once weekly for two weeks; then, it was changed to Dulaglutide 1.5 mg weekly based on insurance formulary. Pioglitazone and bolus insulin dosages were continued, glimeperide was discontinued, and basal insulin was titrated up from 98 units to 160 units at bedtime. He was instructed to use a correction factor of 1 unit for every 50 mg/dL of BG above 150 mg/dL. At baseline, his fasting blood sugar (FBG) and other random times of the day was averaging 300‐400 mg/d/L. Approximately at 50 days after his initial visit with us, his BG started averaging 60‐100 mg/dL and his insulin Aspart (100 μ/mL) dose fell to 20 units before meals and his insulin glargine (300 μ/mL) dose to 100 units at bedtime. After approximately 3 months of follow‐up, the patient reported a home BG averaging 100‐130 mg/dL throughout the day. Toward the end of a 12‐month period, his insulin glargine (300 U/mL) requirements fell to 68 units at bedtime and his insulin Aspart 100 units/mL fell to 15 units before meals. The patient's UACR dropped from 2000 to 887, 382, 410, and 221 mg/g at approximately 3, 6, 9, and 12 months of follow‐up, respectively. It fell from the baseline level of 2000 mg/g to around 82 mg/g at approximately 30 months follow‐up (Figure 1). The eGFR remained stable throughout the follow‐up period (Figure 2). The patient's A1C dropped from the baseline value of 11.4 to 7.4, 8.3, and 8.1 at approximately 3, 6, and 12 months, respectively, and his fructosamine was 219 mmol/L (which correlates to an A1C of 5%‐5.5%) toward the end of the 30 months follow‐up periods. Patient's electrolyte levels were maintained within normal limits throughout the 30 months. Left upper extremity radiocephalic AV fistula was placed in anticipation of possible progression to ESRD requiring hemodialysis (HD). Eight months later, he had a left upper extremity AV brachial cephalic fistula replacing the first left upper extremity brachial basilic. None of the AV fistulas were utilized as he never required HD. There was one instance when patient developed severe diarrhea and vomiting with dehydration due to a viral infection and his eGFR had dropped to 9 ml/min/m^2^ but reverted back to 26 mL/min/m2 within 5 days with adequate hydration. He did discontinue empagliflozin during these 5 days.

### 3.2 Case 2

71‐year‐old Caucasian gentleman with history of T2DM for 7 years presented to endocrinology clinic with baseline A1C of 6.1%, Serum BUN: 28 mg/dL, Serum Creatinine: 2.96 mg/dL, eGFR: 21 mL/min/1.73 m^2^, UACR: 1253 mg/g, NIH Stage 4 CKD and KDIGO Stage G4A3. He was on nateglinide120 mg three times daily. The patient was checking his BG randomly and varied between 120 and 130 mg/dl range. Dapagliflozin 5 mg daily was initiated. Dulaglutide 0.75 mg weekly was also started for the first 4 weeks and then increased to 1.5 mg weekly thereafter with discontinuation of nateglinide within first week of above changes. At 8 weeks follow‐up, the patient's A1C dropped from 6.1% to 5.3%. UACR dropped from 1253 to 159 mg/g (Figure 1). Patient's eGFR, BUN, and serum creatinine were unchanged over the 8 weeks period compared to baseline (Figures 2, 3, 4). At approximately 3 months follow‐up, his fasting BG and prandial BG varied between 100 and 130 mg/dL.

### 3.3 Case 3

A 64‐year‐old pacific islander gentleman with 4‐year history of T2D presented to our clinic with A1C of 13.0%, eGFR: 27 mL/min/1.73 m^2^, Serum BUN: 17 mg/dL, Serum Creatinine: 2.96 mg/d and UACR of 1065.0 mg/g, NIH Stage 4 CKD and KDIGO Stage G4A3. His BG varied from 200 to 220 mg/dL range at different times of the day. The patient was being managed with Insulin Glargine (100 units/mL) 45 units at bedtime and 4‐8 units of Insulin Lispro (U‐100) with each meal. This patient was started on Ertuglflozin 15 mg daily, Dulaglutide 0.75 mg weekly, and his Insulin Glargine was increased to 60 units at bedtime. Dulaglutide dose was then increased to 1.5 mg weekly after 8 weeks. After 3 months of being on the previously mentioned regimen, the patient's A1C decreased from 13% to 6.5% and BP dropped from 148/78 mm Hg to less than 130/80 mm Hg. At 6 months follow‐up; UACR fell from 1065 to 474 mg/g (Figure 1) and eGFR went up from 27 to 52 mL/min/1.73m^2^ (Figure 2). Patient was then started on Metformin 500 mg twice daily. Over the proceeding 8 weeks, the dose of insulin Glargine (100 μ/mL) was gradually decreased to 20 units at bed time to target a fasting blood glucose of 80‐120 mg/dL.

### 3.4 Case 4

74‐year‐old Spanish female patient with history of T2DM for 8 years. At presentation, the patient's A1C was 7.8%, Serum BUN: 37 mg/dL, Serum creatinine: 1.87 mg/dL, eGFR: 26 mL/min/1.73m^2^, UACR: 527 mg/g, NIH Stage 4 CKD and KDIGO Stage G4A3. T2DM in this case was being managed with 40 units of insulin Glargine (100 U/ml) at bedtime, Dulaglutide 1.5 mg weekly and Insulin Lispro (100 U/ml) as per sliding scale (1 unit for 50 points above 150 mg/dL of blood glucose around meal times). Blood glucose readings were around 200 mg/dL throughout the day. This patient was started on Empagliflozin 10 mg daily, the Insulin Lispro (U‐100) was changed to 15 units prior to each meal, and her Insulin Glargine(100 units/ml) was increased to 45 units at bedtime. At the 3 months follow‐up, her blood glucose readings were averaging between 130 and 150 mg/dL and her A1C decreased to 6.6. Her eGFR was maintained at 23 mL/min/1.73 m^2^ and stayed around that level for the entire 15 months of patient follow‐up course (Figure 2). The patient's UACR decreased from 527 at baseline to 92 mg/g at 6 months and continued to drop until the 15 months mark when it was 66 mg/g (Figure 1).

Vital signs (baseline) of cases 1‐4 along with their baseline and follow‐up bicarbonate levels(within normal limits) are shown in Table 1. Electrolytes remained stable during the follow‐up period. Lifestyle modification was emphasized at each follow‐up visit. These cases reported no other adverse events except mild to moderate nausea for the first 4 to 6 weeks attributable to dulaglutide therapy. The observed benefits are above and beyond what is experienced with ACE inhibitor or ARB and a statin as they were already on its stable doses (Table 1) for at least a year prior to initiation of our therapeutic approach.

## 4 DISCUSSION

To the best of our knowledge, positive renal outcomes with safety of concomitant SGLT‐2 inhibitor and GLP1 RA in eGFR below 30 mL/min/m^2^ with macroalbuminuria have not been previously described. Three out of four patients had nephrotic range proteinuria at baseline. None of these patients had end‐stage kidney disease (dialysis, transplantation, or a sustained estimated eGFR of <15 mL/min/1.73 m^2^), a doubling of the serum creatinine level, or death from renal or cardiovascular causes while on these combination therapy. These benefits were seen in patients stratified to have KDIGO Stage G4A3 and therefore very high risk renal patients.

We believe these renal benefits to be a class effect of SGLT‐2 inhibitors and GLP1‐RA. We report the above‐stated benefits with empagliflozin 10 mg in cases 1 and 4 and with dapagliflozin 5 mg and ertugliflozin 15 mg in cases 2 and 3, respectively. All four cases were concomitantly treated with dulaglutide 1.5 mg. The choice of agents was based on insurance coverage/formulary. Possible underlying renoprotective mechanisms are still under investigation but believed to be independent of glycemic control, weight loss, and baseline BMI. Several considerations on the mechanism in which SGLT‐2 inhibitors exert its effect include thrifty substrate hypothesis (ketosis theory), reduction in eGFR triggered by vasoconstriction of afferent renal arteriole from macula densa sensing high distal tubal sodium overload and approximately 3% rise in erythropoiesis thereby reducing the workload of an overloaded/hypoxic kidney. Furthermore, reduction in oxidative stress, serum uric acid, and renal angiotensinogen may also contribute.^5^, ^6^ Renoprotective effects of GLP‐1RA could be due to its anti‐inflammatory effects, vascular endothelium protection,^7^ and amelioration of oxidative stress through the GLP‐1 receptors found on the glomerular, tubular, and the vascular cells of the kidney.^6^, ^7^

In CREDENCE trial, the regression of UACR was approximately 35% (from app.927 mg/g to app. 600 mg/g) at 6 months follow‐up and maintained for a total duration of 2.62 years.^2^ AWARD‐7 trial reported app.23% reduction in UACR with dulaglutide at 6 months in post hoc analysis of patients with eGFR below 30 mL/min/1.73 m^2^.^4^ Here, we report regression of mean UACR of all four cases by 16%, 65%, and 77% at 3, 6, and 9 months, respectively, comparing to baseline. Such impressive and continued reduction in UACR over time could be due to higher baseline UACR and also due to possible synergistic renoprotective effects of simultaneous use of two different class of medications. The mean estimated eGFR in CREDENCE trial ^2^ and DAPA‐CKD trial ^3^ was 56.2 ± 18.2 and app. 43 mL/min/1.73 m^2^, respectively, vs 23.75 ± 1.6 mL/min/1.73 m^2^ in our case series. While these trials reported positive renal outcomes along with its safety with SGLT‐2 inhibitor, we report the same with concomitant GLP‐1RA and SGLT‐2 inhibitor.

Case 1 underwent three different AV shunt placement procedures on three different occasions over a period of 2.5 years in anticipation for possible HD; however, he continues to do well and has not required HD still. The timing of these procedures will have to be modified if such patients are doing well with this strategy. Near doubling of eGFR in case 3 over a period of 6 months is not fully understood; however, we attribute this to impressive A1c and BP reduction along with renoprotective effects of GLP‐1RA and SGLT‐2 inhibitor.

Limitations of our study include retrospective study design and small sample size, but it is an important proof of concept study that will facilitate the design and conduct of large randomized clinical trials to confirm these benefits. Since the initial success of these four patients described here, it has become a standard practice for our clinic to be using SGLT‐2 inhibitors concomitantly with GLP‐1RA in DKD with eGFR below 30 mL/min/1.73 m^2^. Therefore, we shall be able to report longer durability of renal outcomes in larger group of patients in the future.

We conclude that addition of SGLT‐2 inhibitor and GLP‐1RA in high renal risk (KDIGO stage G4A3) T2D patients leads to positive composite end point of slowing the progression to end‐stage kidney disease (dialysis, transplantation, or a sustained estimated eGFR of <15 mL per minute per 1.73 m^2^), a doubling of the serum creatinine level, or death from renal or cardiovascular causes. We recommend that practitioners adopt our approach cautiously with frequent clinical monitoring to ensure similar success. Further randomized clinical studies will be required to confirm the benefits, durability, and possible underlying mechanisms.

## CONFLICT OF INTEREST

NDK serves as faculty member for speaker bureau for BI & Lily & Novo Nordisk.

## AUTHOR CONTRIBUTIONS

NDK: involved in concept and design, execution of the study, wrote first draft of the manuscript and made subsequent revisions. IM: involved in execution of the study and edits.

## ETHICS STATEMENT

NDK and IM are the guarantors of this work and, as such, had full access to all the data in the study and take responsibility for the integrity of the data and the accuracy of the data analysis.

## Data Availability

Data are available on request due to privacy/ethical restrictions.

## References

[1] Lo KB , Gul F , Ram P , et al. The effects of SGLT2 inhibitors on cardiovascular and renal outcomes in diabetic patients: a systematic review and meta‐analysis. Cardiorenal Med. 2020;10(1):1‐10. 31743918 10.1159/000503919

[2] Perkovic V , Jardine MJ , Neal B , et al. Canagliflozin and renal outcomes in type 2 diabetes and nephropathy. N Engl J Med. 2019;380(24):2295‐2306. 30990260 10.1056/NEJMoa1811744

[3] Heerspink HJL , Stefansson BV , Correa‐Rotter R , et al. Dapagliflozin in patients with chronic kidney disease. N Engl J Med. 2020;383(15):1436‐1446. 32970396 10.1056/NEJMoa2024816

[4] Tuttle KR , Lakshmanan MC , Rayner B , et al. Dulaglutide versus insulin glargine in patients with type 2 diabetes and moderate‐to‐severe chronic kidney disease (AWARD‐7): a multicentre, open‐label, randomised trial. Lancet Diabetes Endocrinol. 2018;6(8):605‐617. 29910024 10.1016/S2213-8587(18)30104-9

[5] Ferrannini E , Mark M , Mayoux E . CV Protection in the EMPA‐REG OUTCOME Trial: A "Thrifty Substrate" Hypothesis. Diabetes Care. 2016;39(7):1108‐1114. 27289126 10.2337/dc16-0330

[6] Mudaliar S , Alloju S , Henry RR . Can a shift in fuel energetics explain the beneficial cardiorenal outcomes in the EMPA‐REG OUTCOME Study? A unifying hypothesis. Diabetes Care. 2016;39(7):1115‐1122. 27289124 10.2337/dc16-0542

[7] Dandona P , Ghanim H , Abuaysheh S , et al. Exenatide Increases IL‐1RA Concentration and Induces Nrf‐2Keap‐1regulated antioxidant enzymes: relevance to beta‐cell function. J Clin Endocrinol Metab. 2018;103(3):1180‐1187. 29346597 10.1210/jc.2017-02343

